# Intrauterine, Vacuum-Induced, Hemorrhage-Control Device System Contributing to Blood Loss in Fulminant DIC

**DOI:** 10.1155/crog/3747372

**Published:** 2025-09-03

**Authors:** Alina Zabolotico, Victoria Kucinski, Chad Strittmatter, Bruce Rodgers

**Affiliations:** Department of Obstetrics & Gynecology, Sisters of Charity Hospital, Buffalo, New York, USA

## Abstract

**Background:** Postpartum hemorrhage (PPH) complicates 3%–5% of deliveries. Uterine atony is the most common cause of PPH, and there are several interventions that can be used to improve atony, including uterine massage, uterotonics, and intrauterine balloons. The intrauterine vacuum-induced hemorrhage-control device is a suction device that is placed in the lower uterine segment, which creates negative pressure on the myometrium to induce myometrial contraction.

**Case:** The patient was a 37-year-old G3P1011 female with chronic hypertension who presented for induction of labor at term. Spontaneous rupture of membranes occurred several hours later with simultaneous placental abruption leading to DIC. Urgent cesarean section was performed for fetal distress remote from delivery. Massive transfusion protocol was initiated, and an intrauterine vacuum-induced hemorrhage-control device was placed intravaginally in an attempt to control blood loss. However, rather than acting as a tamponade, the device appeared to draw out a great blood loss with its suction. The patient underwent bilateral uterine artery embolization, and the device was replaced with an intrauterine balloon. Patient was transferred to the ICU, and blood products were transfused until the patient was stabilized.

**Conclusion:** While a preliminary study appeared to show the safety and efficacy of the intrauterine vacuum-induced hemorrhage-control device, its use was limited to uterine hemorrhage secondary to uterine atony. It should be avoided in cases of hemorrhage where coagulopathy is suspected.

## 1. Introduction

One of the most common complications in obstetrics is PPH, complicating approximately 3% to 5% of deliveries. The most common cause is uterine atony, followed by lacerations and coagulopathies. In a term pregnancy, the amount of blood flow to the uterus nears 600 mL/min through the spiral arteries and into the intervillous spaces. When the uterus fails to contract appropriately, uterine atony can lead to massive hemorrhage. When first-line treatments such as uterotonics and uterine massage fail, other mechanisms are used to control bleeding, such as uterine packing, intrauterine balloon, uterine artery ligation, or B-Lynch suture [1–3]. The newly FDA-approved Jada device is a silicone loop lined with 20 vacuum pores [4]. It is placed into the lower uterine segment, and then attached to the low-level wall suction to exert negative pressure on the uterus to induce the myometrium to contract, obliterating the spiral artery lumens [5, 6].

The “PEARLE” study was a single-arm treatment study which appeared to show efficacy and safety of the intrauterine vacuum-induced hemorrhage-control device when used for uterine atony unresponsive to uterotonics. The study did not evaluate the use of Jada in patients with retained placenta, known as maternal coagulopathy, or a quantitative blood loss of more than 1500 mL before device placement [7, 8]. In addition, no randomized controlled trial has yet been undertaken comparing the intrauterine vacuum-induced hemorrhage-control device to existing devices for control of uterine hemorrhage [5, 9, 10]. This is a report of postpartum hemorrhage secondary to DIC in which the intrauterine vacuum-induced hemorrhage-control device was ineffective and may have contributed to increased blood loss.

## 2. Case

The patient was a 37-year-old G3P1011 female presenting to labor and delivery at 37w2d for induction for pre-existing hypertension, maintained on Labetalol 100 mg twice daily. Her pregnancy was complicated by mild polyhydramnios, diet-controlled GDM, and advanced maternal age. She had a history of one previous spontaneous vaginal delivery without complication. The remainder of the patient's history was noncontributory. On admission, her blood pressure was 134/86 mmHg, pulse 87 BPM. FHR was reactive and the tocometer demonstrated rare contractions. Her cervix was 2 cm dilated, 70% effaced, and the fetal head was at −3 station.

Induction was initiated with oxytocin, and an epidural was placed. Following spontaneous rupture of the membranes at 5-cm dilation, tachysystole and recurrent variable decelerations with minimal variability were noted, initially responsive to resuscitative measures including intravenous fluids, discontinuation of oxytocin, and position changes. However, approximately 1 h later, FHR returned to Category 2, characterized by minimal variability and recurrent late decelerations. The patient was repositioned on her hands and knees, at which time bleeding from the epidural site, Foley catheter, and epistaxis were simultaneously noted. There was no evidence of vaginal bleeding. Coagulation studies were obtained, and a cesarean section was initiated for nonreassuring fetal status.

Upon entry into the uterus, several hundred milliliters of blood erupted from the hysterotomy. A viable baby boy with APGAR scores of 1, 5, and 6 at 1, 5, and 10 min, respectively, was delivered without difficulty. Cord gas pH was 7.04, with a base excess of −11.8. The neonatology team was present at the time of delivery, and resuscitative efforts were undertaken immediately, including intubation. The placenta was > 50% abrupt. After the delivery of the placenta, the hysterotomy was closed. There was minimal bleeding noted at this time, but blood appeared water-like, without the formation of clots. As closure of the abdomen continued, hemostasis became difficult to achieve as the patient bled from every puncture site created.

Massive transfusion protocol was initiated, and patient was transfused with packed red blood cells (pRBCs) and fresh frozen plasma (FFP) intraoperatively. Intraoperative labs revealed hemoglobin 8.4 g/dL, platelets 97/*μ*L, creatinine 0.99 mg/dL, fibrinogen < 35 g/L, PT 28.7 s, PTT 38.5 s, and INR 2.4. Surgery was completed with adequate intra-abdominal hemostasis, and attention was turned to the patient's vaginal bleeding. The fundus was firm and below the umbilicus with adequate tone, but the patient was losing approximately 100 mL of blood each time the fundus was palpated. Total blood loss at this time was estimated to be 3000 mL. The decision was made to proceed with placement of intrauterine vacuum-induced hemorrhage-control device transvaginally per manufacturer's instructions with the active portion in place in the lower uterine segment and the vaginal balloon flush against the cervix and inflated with 120 mL of sterile saline. The device was attached to 80 mg Hg of wall suction and immediately, several hundred milliliters of blood poured into the suction canister. It appeared that the Jada was exacerbating the patient's blood loss. Removal of the device was considered; however, the patient was instead transferred to a different unit for uterine artery embolization (UAE). The decision was made to leave the intrauterine vacuum-induced hemorrhage-control device in place during transfer; however, it was rapidly replaced with an intra-uterine balloon for better tamponade once the patient was relocated. There was an additional 3200 mL of blood loss recorded since the insertion of the intrauterine vacuum-induced hemorrhage-control device. UAE was completed bilaterally. While undergoing the procedure, patient was profoundly hypotensive and hypoxic with BP readings 50/30s and SPO_2_ between 60% and 70%, leading to intubation. Labs obtained during the UAE were as follows: hemoglobin 3.4 g/dL, platelets 78/*μ*L, creatinine 1.23 mg/dL, fibrinogen 83 g/L, PT 19 s, PTT 67.2 s, INR 1.6, lactic acid 18.3 mg/dL, and pH < 6.95. Vaginal bleeding was slightly improved following UAE.

Postoperatively, the patient appeared more alert and was extubated. She was transferred to the intensive care unit (ICU) and ultimately received: 12 units pRBCs, 10 units FFP, 4 units of cryoprecipitate, and 3 units of platelets following a total blood loss of 8255 mL. Quantitative blood loss was measured by weighing lap sponges and chux pads and assessing the blood in the suction canisters. Intrauterine balloon was removed on postoperative Day 1 with minimal bleeding. Her postpartum course was complicated by a postoperative ileus, shock liver, and acute kidney injury requiring dialysis. Her liver function enzymes normalized after several days, but creatinine remained elevated. The patient was dialysis-dependent upon discharge. However, 2 months postoperatively, she had full return of kidney function.

## 3. Discussion

This case report demonstrated the lack of efficacy of the intrauterine vacuum-induced hemorrhage-control device system in a patient with postpartum hemorrhage due to DIC. The intended use of the intrauterine vacuum-induced hemorrhage-control device is for abnormal uterine bleeding in the setting of uterine atony; it is not intended for PPH caused by coagulopathy. The intrauterine vacuum-induced hemorrhage-control device was utilized on this patient with PPH not with the intention to correct atony, but rather with the intent that suction would provide enough negative pressure to compress the spiral arteries and achieve hemostasis. Unfortunately, rather than decreasing bleeding, insertion of the device had seemed to pull blood from the patient instead, with continued heavy output into the suction canister through the tubing system.

We theorize that the viscosity of the blood with this degree of coagulopathy was not amenable to the degree of compression placed on the spiral arteries. In addition, in the presence of coagulopathy, the negative intrauterine pressure generated by the device may have increased blood loss from the spiral arteries. While there is no definitive evidence that the intrauterine vacuum-induced hemorrhage-control device contributed to this patient's blood loss, we raise the concern that coagulopathies are not currently listed as a contraindication to the use of the intrauterine vacuum-induced hemorrhage-control device. If such patients were excluded from the PEARLE study for either concern of lack of efficacy or concern for clinical deterioration, then it should also be included on the list of contraindications for these same reasons. DIC is not listed as an absolute contraindication for the use of Jada. The purpose of this case report is to make providers who are not familiar with Jada aware of its limitations in the setting of DIC.

The implication of this case report is that the Jada may have exacerbated the hemorrhage. The Jada device is not contraindicated in DIC, per se, but extreme caution is required, and it is not a primary treatment for coagulopathy associated with fulminant DIC. The intrauterine vacuum-induced hemorrhage-control device is a revolutionary tool to safely and effectively treat PPH secondary to uterine atony. Further studies need to be done to evaluate if it has limitations in managing hemorrhages of a different nature.

## Data Availability

Data sharing not applicable to this article as no datasets were generated or analysed during the current study.

## General Statement

*Teaching Points*: When uterine hemorrhage is occurring in the face of a well-contracted uterus, coagulopathy should be suspected. In such cases, intrauterine vacuum-induced hemorrhage-control devices would not be expected to be effective and potentially could worsen blood loss.

Until such time as the efficacy of the intrauterine vacuum-induced hemorrhage-control device is studied for other indications, its use should be confined to patients with postpartum hemorrhage due to uterine atony.
